# Immunologic Response to Tumor Ablation with Irreversible Electroporation

**DOI:** 10.1371/journal.pone.0048749

**Published:** 2012-11-06

**Authors:** Xiaoxiang Li, Kui Xu, Wei Li, Xiuchun Qiu, Baoan Ma, Qingyu Fan, Zhao Li

**Affiliations:** Orthopedics Oncology Institute of Chinese PLA, Tangdu Hospital, the Fourth Military Medical University, Xi’an, Shannxi, China; West Virginia University, United States of America

## Abstract

**Background:**

Irreversible electroporation (IRE) is a promising technique for the focal treatment of pathologic tissues, which involves placing minimally invasive electrodes within the targeted region. However, the knowledge about the therapeutic efficacy and immune reactions in response to IRE remains in its infancy.

**Methods:**

In this work, to detect whether tumor ablation with IRE could trigger the immunologic response, we developed an osteosarcoma rat model and applied IRE directly to ablate the tumor. In the experiment, 118 SD rats were randomized into 4 groups: the control, sham operation, surgical resection, and IRE groups. Another 28 rats without tumor cell implantation served as the normal non-tumor-bearing group. We analyzed the changes in T lymphocyte subsets, sIL-2R and IL-10 levels in the peripheral blood one day before operation, as well as at 1, 3, 7,14 and 21 days after the operation. Moreover, splenocytes were assayed for IFN-γ and IL-4 production using intracellular cytokine staining one day before the operation, as well as at 7 and 21 days after operation.

**Results:**

We found that direct IRE completely ablated the tumor cells. A significant increase in peripheral lymphocytes, especially CD3^+^ and CD4^+^ cells, as well as an increased ratio of CD4^+^/CD8^+^ were detectable 7 days after operation in both the IRE and surgical resection groups. Compared with the surgical resection group, the IRE group exhibited a stronger cellular immune response. The sIL-2R level of the peripheral blood in the IRE group decreased with time and was significantly different from that in the surgical resection group. Moreover, ablation with IRE significantly increased the percentage of IFN-γ-positive splenocytes.

**Conclusion:**

These findings indicated that IRE could not only locally destroy the tumor but also change the status of cellular immunity in osteosarcoma-bearing rats. This provides experimental evidence for the clinical application of IRE in osteosarcoma treatment.

## Introduction

As alternatives to surgical resection, minimally invasive tumor ablation therapies such as radiofrequency, laser, microwave and cryoablation have been developed for the treatment of benign or malignant tumors, and these techniques can be used to ablate undesirable tissue in a well-controlled and precise way [1]–[3]. Most of these therapies are based on thermal ablation techniques that destroy the tumor tissue by increasing or decreasing temperatures to induce irreversible cellular injury. Recently, irreversible electroporation (IRE) has begun receiving attention as a relative newcomer to the field of tumor ablation techniques in focal treatment. IRE is used to apply short length but high voltage electrical pulses to the cell, generating a destabilizing electric potential and causing the formation of permanent nanoscale defects in the cell membrane. The permanent permeability of cell membrane leads to changes in cell homeostasis and cell death [4], [5]. IRE lacks many of the drawbacks of other conventional thermal ablation methods, including tumor protection next to large vessels due to a heat sink effect and the associated destruction of normal structures [6]. Our previous research also indicated that nerves treated with IRE can attain full recovery [7]. Many encouraging results have been reported in the IRE treatment of solid tumors in humans, including lung, prostate, kidney, and liver cancers [8]–[10]. Human treatment has revealed that IRE is a feasible and safe technique that could offer some potential advantages over current thermal ablation techniques.

It is thought that IRE achieves focal tumor ablation by destabilizing the cell membrane and inducing cell death in a non-thermal manner. Thus, many autologous tumor-antigens will remain in situ after IRE treatment, and there remains a question of whether IRE of solid tumors could evoke an immune response. The only immunohistochemical study focusing on immune response to tumor ablation with IRE used immunohistochemistry to show that there was no recruitment of immune cells such as CD4^+^, CD8^+^ T lymphocytes, macrophages, activated antigen presenting cells (APCs) and dendritic cells after 72 hours [11]. However, many other aspects of immune responses, such as changes in cytokines peripheral and intratumoral immune cell subsets and specific antitumor activity remain to be clarified. In this study, we aimed to explore the immunologic response to tumor ablation with IRE using a subcutaneously xenotransplanted osteosarcoma model in rats and to provide experimental evidence supporting the clinical application of this technique for osteosarcoma treatment.

## Materials and Methods

### Cell Lines, Animals and Tumors

All the experimental protocols involving animals were reviewed and approved by the Ethics Committee of Tangdu Hospital, Fourth Military Medical University (approval ID:2011A028). Male Sprague-Dawley (SD) rats, 2- to 3-weeks old, were purchased from the laboratory animal research centre of the Fourth Military Medical University (FMMU). The UMR106 osteosarcoma cell line was purchased from the American Type Culture Collection (ATCC; Manassas, VA, USA). The UMR106 cells were cultured in Dulbecco's Modified Eagle's Medium (Hyclone) containing 10% FBS (Sigma), 100 U/ml penicillin and 100 µg/ml streptomycin, confirmed to be mycoplasma-free by routine testing, in the presence of 5% CO_2_ in a humidified incubator. Tumor cells were washed with 0.01 mmol/L phosphate buffer solution (PBS, pH = 7.4) twice and then resuspended in PBS at a density of 2×10^7^/ml. Under sterile conditions, 0.5 ml of the cell suspension was slowly injected into the subcutaneous tissue on the back of five rats. At 20 days after injection, the largest tumors were selected for primary culture. The process was repeated, and the tumor development rate was 100% after two rounds of screening. The diameter of the tumors reached nearly 1.0 centimeters at 8–12 days after transplantation. After the screening of the tumor cells, each of 118 SD rats was injected subcutaneously with 0.5 ml of the cell suspension at a density of 2×10^7^/ml on the back, while 28 SD rats were injected with PBS for the non-tumor-bearing control group.

### Animal Grouping and Treatment

When the diameter of the tumors reached nearly 1.0 centimeters, the rats were randomized into 4 groups: the control group (n = 28), sham operation group (n = 28), surgical resection group (n = 28) and IRE group (n = 34). Another 28 rats without tumor cell implantation were analyzed as the normal non-tumor-bearing group.

**Figure 1:**
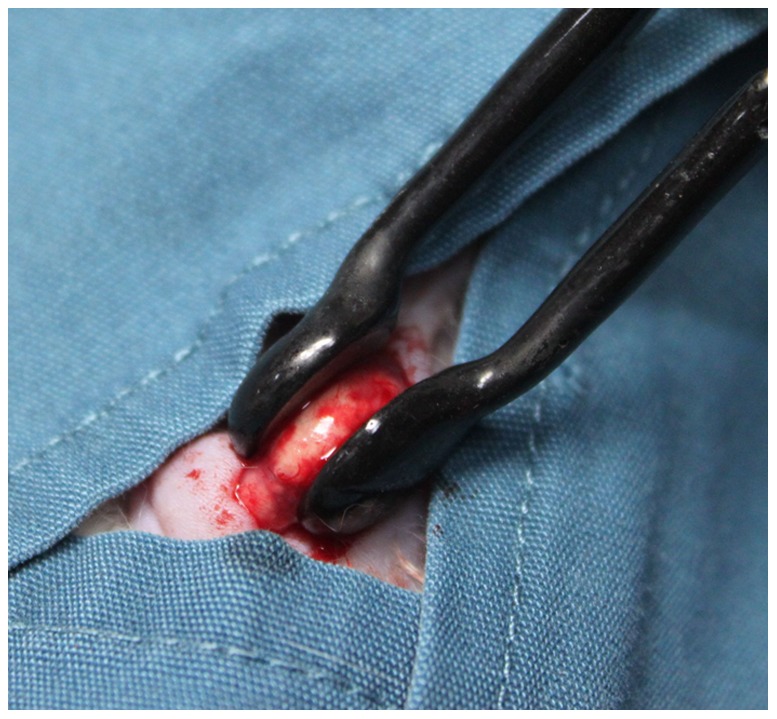
The IRE device clamping the tumor in the rat.

For the IRE group, the animals were anaesthetized by an intraperitoneal injection of sodium pentobarbital (10 mg/ml, 40 mg/kg body weight). A small incision was made on the skin near the tumor, and particular care was exercised to avoid cutting the main blood vessels nourishing the tumor. A specially designed hand-held clamp containing two parallel metal electrodes (Tweezertrodes, BTX, MA, USA) was placed in direct contact with both sides of the subcutaneous tumor with the tumor sandwiched between the parallel plates to accurately control the electric field amplitude and distribution in the tumor tissue (**Fig. 1**). Good contact of the electrodes with the tumor tissue was produced using an electrocardiography paste that had been sterilized by ^60^Co γ-irradiation. The distance between the electrodes was measured with a caliper, and then the pulse generator was set to deliver an approximate applied electrical field of 1500 V/cm. We delivered 9 trains of 10 direct current square pulses, each 100 µs long, between the electrodes using an electroporation pulse generator (TP3032, Teslaman, Dalian, China). The electrodes were rotated 90° between each train of pulses, and a total of 90 square pulses were delivered. In the surgical resection group, the tumor was exposed and completely resected without IRE. In the sham operation group, the plate electrodes were placed in direct contact with the tumor mass but not used for IRE. The rats in the control group and normal non-tumor-bearing group received no particular interventions. All the operations were performed under strictly sterile conditions. After the operations, the incision was routinely closed, and the rats were kept separately in normal housing conditions. All surgeries were performed by the same surgeon.

### T lymphocyte Cell Subset Analysis

In each of the groups, 0.1 µl anticoagulated venous whole blood was procured from the rats 1 day before the operation, as well as at 1, 3, 7, 14 and 21 days after the operation. Fluorescently labeled CD3^+^ (Clone: 1F4, Becton Dickinson, CA, USA), CD4^+^ (Clone: OX-38, BD) and CD8^+^ (Clone: OX-8, BD) monoclonal antibodies were added to test tubes containing 100 µl blood samples. After gentle shaking, all tubes were placed at room temperature for 15 minutes, and then mixed with 1 ml hemolysin. The tubes were kept in darkness at room temperature for 15 minutes and centrifuged at 5000 r/min for 5 minutes. The supernatant was removed, and the cells were washed twice. Then, 500 µl PBS was added to each tube, and the cells were fully resuspended by gentle shaking. Flow cytometry was used to determine the percentages of the CD3^+^, CD4^+^ and CD8^+^ cell subsets in peripheral blood.

### Flow Cytometry for Cytokine Profile Analysis

Six rats were killed independently in every group at three different timepoints (1 day before operation and at 7 and 21 days after operation), and their splenocytes were removed aseptically. Fat and some other non-spleen tissue was removed carefully. Splenocytes procured from each rat were prepared with 2×10^6^/ml in the same way. 1 ml spleen cell suspension was used for analysis with stimulant. A PMA/Ionomycin mixture (PMA 5 ng/ml + Ionomycin 500 ng/ml, MultiSciences, Hangzhou, China) and monensin (2 µM, eBioscience, San Diego, CA, USA) were added to the cell suspensions. Then, the cells were incubated for 6 hours at 37°C. After gentle shaking, the cells were kept at room temperature for 10 minutes and then mixed with 2 ml hemolysin. The tubes were set aside for 15 minutes and then centrifuged at 5000 r/min for 15 minutes. The supernatant was removed, and the cell suspensions were incubated with fixation buffer at 4°C overnight. Then, the cells were washed twice in 2 ml permeabilization buffer and centrifuged at 5000 r/min for 15 minutes, followed by the addition of fluorescently labeled IFN-γ (Clone: DB-1, Biolegend, San Diego, CA, USA) and IL-4 (Clone: OX-81, Biolegend) monoclonal antibodies and placed in the dark at room temperature for 30 minutes. The cells were then washed twice and then subjected to flow cytometry to ascertain the percentages of IFN-γ and IL-4 cell subsets.

### Serologic Examination

ELISA was used to measure the serum sIL-2R and IL-10 levels in 100 µl samples taken 1 day before the operation and at 1, 3, 7, 14 and 21 days after the operation in all five groups.

### Statistical Analysis

The data were expressed as means ± standard deviations. Significant differences between timepoints or groups were analyzed using ANOVA for repeated measures with Tamhane’s T2 method for multiple comparisons in SPSS 17.0 (SPSS, Chicago, IL, USA). Differences were considered statistically significant when P<0.05.

## Results

### Rat Survival

After inoculation with UMR106 osteosarcoma cells, the volume of the tumor mass increased gradually. The tumors reached nearly 1.0 centimeters in diameter at 6–9 days after the inoculation, but none of the rats died due to tumor growth during the experiment. In the IRE group, the tumor volume tended to decrease gradually after the operation. No in-situ tumor recurrence was found in the surgical resection group or in the IRE group.

### Hematoxylin–Eosin (HE) Staining Detection

**Figure 2:**
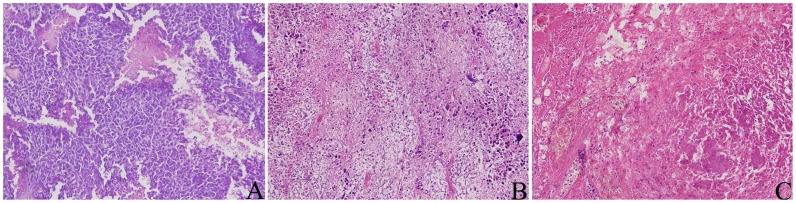
Hematoxylin and eosin staining of the tumor tissues. (A) 1 day prior to the IRE operation, the tumor cells displayed a large nucleus surrounded by a well marked cytoplasm and a well defined cell membrane; (B) 1 day after IRE, obvious tissue necrosis appeared; (C) 3 days after IRE, a continued increase in cellular eosinophilia, vascular congestion and inflammatory cell infiltration was observed (×200).

Histological examination of the tissue taken from the site of tumor implantation was performed by a pathologist. Nine tumors in the IRE group were examined 1 day before IRE, as well as at 1 and 3 days after IRE, respectively. As shown in **Fig. 2A**, 1 day before IRE, the tumor cells displayed a large nucleus surrounded by well-marked cytoplasm and a well-defined cell membrane. On 1 day after IRE (**Fig. 2B**), obvious tissue necrosis appeared. HE staining showed areas of extensive and severe cell death, with pyknotic hyperchromatic nuclei and eosinophilic cytoplasm. Meanwhile, vascular congestion and inflammatory cell infiltration was observed. At 3 days after IRE, there was a continued increase in cellular eosinophilia, with significant necrosis and inflammation of the ablation zone. No viable tumor cells were observed in the IRE-ablated area. Complete cell death was achieved in the targeted tumor tissue (**Fig. 2C**).

### T lymphocyte Subset Changes

**Figure 3:**
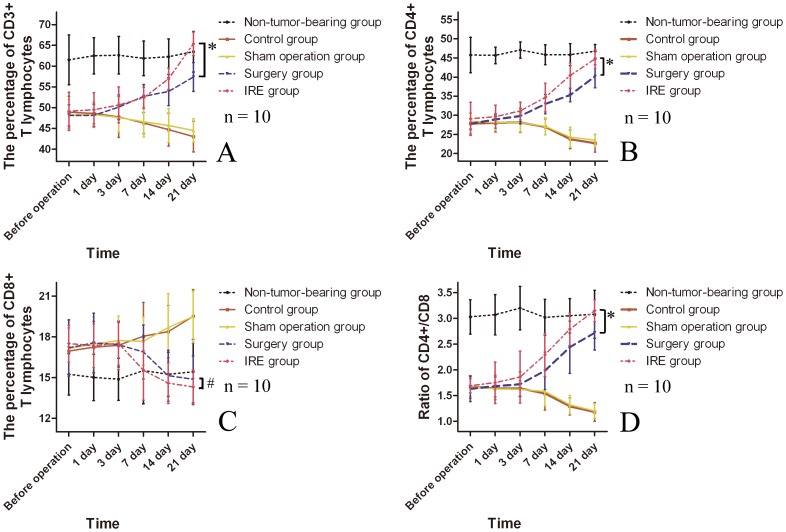
Changes in T lymphocyte subset percentage (A, B, C) and CD4+/CD8+ ratio (D). *p<0.05; #p>0.05.

Compared with the non-tumor-bearing group, the percentages of CD3^+^ T lymphocytes, CD4^+^ T lymphocytes and the CD4^+^/CD8^+^ ratio of tumor-bearing rats were significantly lower before operation (P<0.05) (**Fig. 3**). The percentages of CD3^+^ and CD4^+^ cells and the CD4^+^/CD8^+^ ratio greatly increased 7 days after operation in both the surgical resection group and IRE group and were significantly different from those in sham operation group and control group. Moreover, in the IRE group, the percentages of CD3^+^ and CD4^+^ and the CD4^+^/CD8^+^ ratio increased more significantly than those in the surgical resection group 21 days after operation (P<0.05). Moreover, there were no differences in the percentages of CD3^+^ T lymphocytes and CD4^+^ T lymphocytes at 21 days after operation between the non-tumor-bearing group and the IRE group, and the ratio of CD4^+^/CD8^+^ in the IRE group was higher than that in non-tumor-bearing group, although this difference was not statistically significant (P>0.05).

Compared with the non-tumor-bearing group, tumor-bearing rats showed higher percentages of CD8^+^ T lymphocytes before operation, but this difference was not statistically significant (P>0.05). The percentages of CD8^+^ T lymphocytes in the surgical resection group and the IRE group decreased greatly 14 days after the operation and were significantly different from those in the sham operation group and the control group. However, comparing the surgical resection group, IRE group and non-tumor-bearing group, we found that there were no significant differences between any two groups in the percentages of CD8^+^ T lymphocytes at 14 or 21 days after operation.

### Cytokine IFN-γ-Positive and IL-4-Positive Splenocyte Analysis

Splenocytes were assayed for IFN-γ and IL-4 production using intracellular cytokine staining.

**Figure 4:**
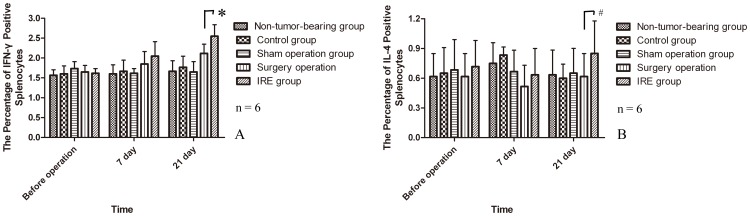
Changes in cytokine IFN-γ-positive (A) and IL-4-positive (B) splenocytes.

There were no significant differences in the percentage of IFN-γ-positive splenocytes among the five groups before operation (P>0.05) **(** **Fig. 4**). The percentage of IFN-γ-positive splenocytes greatly increased with time in the surgical resection group and IRE group, and it was significantly higher than that in the other three groups at 21 days after operation. Furthermore, the IRE group showed a significantly higher percentage of IFN-γ-positive splenocytes than did the control group and surgical resection group. However, the percentage of IL-4-positive splenocytes remained similar in all five groups throughout the experiment.

### Serum sIL-2R and IL-10

**Figure 5:**
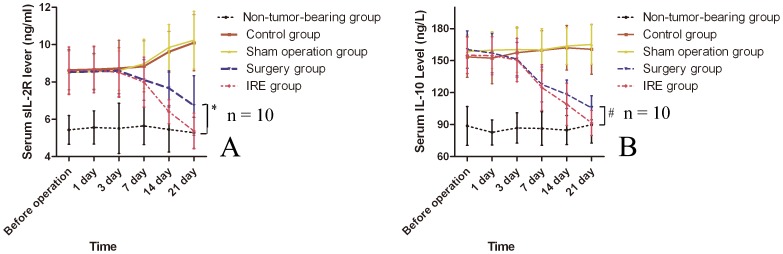
Changes in the serum sIL-2R (A) and IL-10 (B) levels of peripheral blood. *p<0.05; #p>0.05.

Tumor-bearing rats showed significantly higher serum levels of both sIL-2R and IL-10 than did non-tumor-bearing rats prior to the operation (P<0.05) (**Fig. 5**). The sIL-2R and IL-10 levels decreased with time in the surgical resection group and IRE group, and these values were significantly different from those in the sham operation group and control group 7 days after operation. Furthermore, the serum sIL-2R level in the IRE group decreased more rapidly than did that in the surgical resection group from 14 to 21 days after operation (P<0.05). Until 21 days after operation, there was no significant difference in the serum sIL-2R level between the IRE group and the non-tumor-bearing group. However, no significant difference in serum IL-10 level was found between the IRE group and the surgical resection group 14 days after operation, and these values were similar to those in the non-tumor-bearing group at 21 days after operation.

## Discussion

In the present study, we developed an osteosarcoma animal model to evaluate the effect of tumor ablation with IRE on cellular immunity. Because we wanted to detect the cellular immune response after tumor ablation, immunodeficient animals were not suitable for our experiments. Our colleagues’ previous study established a reproducible model of femur osteosarcoma in the rat [12], but the location of the tumor in that model was not suitable for the IRE operation. Furthermore, due to the complexity of the tumor anatomy, it is impossible to ensure complete removal of the tumor. In the study, after two rounds of screening of UMR106, although at least 10^7^ cells had to be transplanted, the reproducible stability of the subcutaneous injection technique to establish an osteosarcoma-bearing model was satisfied, and the oncogenic rate was 100%. In our experiment, we found that the application of 1500 V/cm in 9 trains of 10 direct current square pulses, each 100 µs long, could produce complete osteosarcoma cell ablation after IRE treatment.

CD3^+^ T lymphocytes represent the major lymphocyte subset in peripheral blood, and T cell-mediated immune responses represent the major source of cellular antitumor immunity in cancer patients [13]. T lymphocytes are divided into CD4^+^ (T helper cells) and CD8^+^ subsets (T suppressor/cytotoxic cells), and the CD4^+^/CD8^+^ ratio is linked to T lymphocyte-mediated function. In clinical practice, the CD4^+^/CD8^+^ ratio is generally used as an indicator of antitumor immunity [14] and as a prognostic flag for cancer patients receiving immunomodulative therapy [15]. They are often used to evaluate the immunologic response to tumor ablation with thermal ablation, such as radiofrequency and cryoablation [16]–[18]. In the present study, we found that the percentages of CD3^+^ T lymphocytes and CD4^+^ T lymphocytes, as well as the CD4^+^/CD8^+^ ratio, of tumor-bearing rats was lower than that in the non-tumor-bearing group before operation. Both surgical tumor resection and IRE treatment reduced the percentage of CD8^+^ T lymphocytes in tumor-bearing rats, but there was no statistically significant difference between the two groups. The percentages of CD3^+^ T lymphocytes and CD4^+^ T lymphocytes as well as the CD4^+^/CD8^+^ ratio of the surgical resection group and IRE group increased after operation (P<0.05), and those in IRE group increased more rapidly. Such changes were even more prominent at 14 and 21 days after operation. However, the indexes were similar in the IRE group and surgical resection group 7 days after operation (P>0.05). At 21 days after IRE treatment, the rats in the IRE group had similar percentages of CD3^+^ and CD4^+^ cells and a similar CD4^+^/CD8^+^ ratio compared with the non-tumor-bearing rats. This result demonstrated that surgical resection could remove the tumor tissue but not evoke a great immune response, while the increased percentage of CD4^+^ T helper cells and relatively stable percentage of CD8^+^ suppressor T lymphocytes following IRE treatment could give rise to the increased CD4+/CD8+ cell ratio, suggesting an enhancement in host immunity after IRE treatment. In most malignant diseases, elevated levels of serum sIL-2R are observed [19], [20]. Serum sIL-2R is a useful parameter for evaluating disease stage and monitoring the disease progression during post-treatment follow-up [21], [22]. In this study, we also found that the serum sIL-2R (soluble interleukin-2 receptor) level in the peripheral blood exhibited the same change as the T lymphocytes. This result indicated that the immune response was strengthened after tumor ablation with irreversible electroporation. IL-10 is a multifunctional cytokine with both immunosuppressive and anti-angiogenic functions, and it may have both tumor-promoting and -inhibiting properties [23], [24]. It was found to be a more powerful outright inhibitor of T-helper 1 T cells (Th1) functions than IL-4 [25]–[27]. In our study, the IL-10 level decreased with time in the surgical resection group and the IRE group, and it was significantly different from those in the sham operation group and the control group. However, there was no significant difference in the serum IL-10 levels of the IRE group and the surgical resection group. This indicated that IRE treatment, like tumor resection, could release the immunosuppression caused by high IL-10.

Furthermore, it is known that T-cells exert their effector functions partly by producing and releasing cytokines. Th1 and Th2 cells are characterized by their distinct cytokine expression patterns. Th1 cells secrete IFN-γ and IL-2, whereas Th2 cells produce IL-4, IL-5 and IL-10 [28]. A cytokine profile analysis of the percentage of IFN-γ and IL-4-positive splenocytes showed that there was no statistically significant difference between the five groups before operation. The percentage of IFN-γ-positive splenocytes distinctly increased in the IRE group after the treatment, while IL-4 remained constant. These results might indicate there was no significant Th1/Th2 shift in tumor-bearing animals prior to the operation, while Th1-induced cellular immunity was changed more greatly than Th2-induced humoral immunity 21 days after IRE. Although the mechanism underlying this complex change is not yet clear, these results indicate that IRE could change the status of cellular immunity of subcutaneously xenotransplanted osteosarcoma-bearing rats.

In the past few decades, thermal ablation, in which high or low temperatures are applied to evoke protein denaturation, tissue necrosis and tumor destruction, has played an important role in the treatment of patients who cannot undergo surgical resection [1]–[3]. Spontaneous distant tumor regression after thermal ablation has been reported in several cases, suggesting the possibility that immune reactions may be induced by thermal ablation [29], [30]. Although the involved mechanisms have not yet been thoroughly studied, several key observations have been made: thermal ablation-induced necrosis may naturally coordinate both adaptive and innate immunity by (1) causing local inflammation [31]; (2) motivating the recruitment and activation of immune effector cells nearby and presumably inside the damaged tumor tissue [32]–[34]; (3) activating antitumor adaptive immunity and antibody production, which can help local tumor elimination, control distant tumors including micrometastases, and establish enduring antitumor immunological memory [32], [33], [35]–[37]. Moreover, the depletion of Tregs attributed to the removal of tumor tissue may mostly overcome local immunosuppression, smoothly transitioning towards effective antitumor immunity [38], [39] (reviewed by Haen S. et al. elsewhere [40]).

Induced immune responses, however, are generally weak and likely insufficient for the complete eradication of established tumors and durable prevention of disease progression [32], [41]. Except cryosurgery, all the above-mentioned techniques use hyperthermia. Hyperthermia would cause the melting and fusing of cell membranes as well as protein denaturation, in which proteins are transformed from their native state to a more random state of lower organization. The unfolding of the three dimensional protein structure can destroy the structure of antigenic determinants [31], [42]. This means that little of the remaining tumor debris are potential sources of tumor-associated antigens available to stimulate the immune system. On the other hand, thermal ablation induces coagulative necrosis of tumor tissue as well as the vascellum [42], [43]. It is difficult for immune cells to infiltrate through blood vessels. Unlike thermal ablation, IRE plays a role in ablating tumor cells in a non-thermal manner. It is thought that the permanent disruption of the lipid bilayer integrity caused by IRE could allow the exchange of intra- and extracellular components via nano-size pores and cause irreversible damage to cellular homeostasis [44]–[46]. This disruption of the cellular membrane, which is very different from that in thermal ablation, is the key mechanism for cell death. This means that more intact tumor proteins and tumor-associated antigens could be available to stimulate the immune system. Our previous studies have shown that minimal endothelial damage in small vessels can be observed in the early phase after IRE treatment but that the damaged vessels become normal with an intact endothelium 3 weeks after IRE treatment [7]. The repaired blood vessels may provide an effective route for APCs to reach the ablation area. All of these observations suggest that ablation with IRE is most likely more effective in immunomodulation than is thermal ablation. Al-Sakere B et al. tried to study immune cell recruitment during the treatment of sarcoma in mice with IRE by immunohistochemistry [11]. However, they did not observe infiltration by immune cells (CD4^+^, CD8^+^ T lymphocytes, macrophages, activated antigen presenting cells and dendritic cells) at 72 hours after ablation, and they tended to attribute that to the destruction of the vascellum. However, as in many other studies, obvious immunocyte infiltration was found in ablation areas after IRE treatment in the present study [47]–[50], whereas no authors other than Al-Sakere B et al. performed immunohistochemical studies. The different results may be ascribed to the variety of possible immunocytes, such as neutrophils and plasma cells [32]–[34], [51]. To the best of our knowledge, there have been no other reports focusing on or correlating the effect of tumor ablation with IRE to cellular immunity. Our results show that IRE could effectively reduce tumor load and change the status of cellular immunity in osteosarcoma-bearing rats.

Recent advances in genomics, proteomics and immunology have stimulated the clinical development of numerous cancer immunotherapies by directing the patients’ own immune systems to destroy tumor cells. More and more studies emphasize a comprehensive treatment incorporating surgery, chemotherapy, radiotherapy and immunotherapy in the treatment of malignant tumors. Despite significant progress, there is still much room for improvement in the overall survival of cancer patients. For example, in the last 20 years, despite advances in diagnostic imaging, the evolution of neoadjuvant chemotherapy and the refinements in limb-salvage surgery, the progression-free survival rate remains poor for patients with metastatic, recurrent or unresectable osteosarcoma. This situation has remained relatively unchanged [52], [53]. Our present study demonstrated that, in addition to the local destruction of the tumor, ablation with IRE could also most likely change the status of cellular immunity, which could be potentially applied in tumor treatment to improve patient prognosis.

However, the present study represents only preliminary research on the effect of tumor ablation with IRE on cellular immune response. No experiments were performed to provide evidence of the tumor-specificity of the observed change in cellular immunity, and the mechanisms involved are far from thoroughly clear. Thus, more studies should be performed to clarify the mechanisms of the immune response caused by tumor ablation with IRE, such as whether the response is tumor-specific or can play a protective role, how long these effects could last and so on. Cytotoxicity assays and rechallenge of successfully treated rats will be important experiments to confirm that specific antitumor immunity is caused by IRE.

In conclusion, we developed an animal model to evaluate the immune response caused by tumor ablation with IRE. The results demonstrated that in addition to the local destruction of tumor tissue, ablation with IRE could also change the status of cellular immunity in osteosarcoma-bearing rats. These results provide experimental evidence supporting the clinical application of tumor ablation with IRE for osteosarcoma treatment.

## References

[1] Gillams AR, Lees WR (2008) Five-year survival following radiofrequency ablation of small, solitary, hepatic colorectal metastases. J Vasc Interv Radiol 19: 712–717. 18440460 10.1016/j.jvir.2008.01.016

[2] Callstrom MR, Atwell TD, Charboneau JW, Farrell MA, Goetz MP, et al (2006) Painful metastases involving bone: percutaneous image-guided cryoablation–prospective trial interim analysis. Radiology 241: 572–580. 17057075 10.1148/radiol.2412051247

[3] Goldberg SN, Grassi CJ, Cardella JF, Charboneau JW, Dodd GD, 3rd, et al (2009) Image-guided tumor ablation: standardization of terminology and reporting criteria. J Vasc Interv Radiol 20: S377–390. 19560026 10.1016/j.jvir.2009.04.011

[4] Rubinsky B, Onik G, Mikus P (2007) Irreversible electroporation: a new ablation modality–clinical implications. Technol Cancer Res Treat 6: 37–48. 17241099 10.1177/153303460700600106

[5] Maor E, Ivorra A, Leor J, Rubinsky B (2007) The effect of irreversible electroporation on blood vessels. Technol Cancer Res Treat 6: 307–312. 17668938 10.1177/153303460700600407

[6] Lee EW, Thai S, Kee ST (2010) Irreversible electroporation: a novel image-guided cancer therapy. Gut Liver 4 Suppl 1 S99–S104. 21103304 10.5009/gnl.2010.4.S1.S99 PMC2989557

[7] Li W, Fan Q, Ji Z, Qiu X, Li Z (2011) The effects of irreversible electroporation (IRE) on nerves. PLoS One 6: e18831. 21533143 10.1371/journal.pone.0018831 PMC3077412

[8] Pech M, Janitzky A, Wendler JJ, Strang C, Blaschke S, et al (2011) Irreversible electroporation of renal cell carcinoma: a first-in-man phase I clinical study. Cardiovasc Intervent Radiol 34: 132–138. 20711837 10.1007/s00270-010-9964-1

[9] Ball C, Thomson KR, Kavnoudias H (2010) Irreversible electroporation: a new challenge in “out of operating theater” anesthesia. Anesth Analg 110: 1305–1309. 20142349 10.1213/ANE.0b013e3181d27b30

[10] Thomson KR, Cheung W, Ellis SJ, Federman D, Kavnoudias H, et al (2011) Investigation of the safety of irreversible electroporation in humans. J Vasc Interv Radiol 22: 611–621. 21439847 10.1016/j.jvir.2010.12.014

[11] Al-Sakere B, Bernat C, Andre F, Connault E, Opolon P, et al (2007) A study of the immunological response to tumor ablation with irreversible electroporation. Technol Cancer Res Treat 6: 301–306. 17668937 10.1177/153303460700600406

[12] Yu Z, Sun H, Fan Q, Long H, Yang T, et al (2009) Establishment of reproducible osteosarcoma rat model using orthotopic implantation technique. Oncol Rep 21: 1175–1180. 19360291 10.3892/or_00000338

[13] Whiteside TL, Heberman RB (2003) Effectors of immunity and rationale for immunotherapy. In: Donald WK, Raphael EP, Ralph RW, Robert CB, Ted SG et al.., editors. Holland-Frei Cancer Medicine. 221–228.

[14] Mafune K, Tanaka Y (2000) Influence of multimodality therapy on the cellular immunity of patients with esophageal cancer. Ann Surg Oncol 7: 609–616. 11005560 10.1007/BF02725341

[15] Hernberg M, Muhonen T, Turunen JP, Hahka-Kemppinen M, Pyrhonen S (1996) The CD4+/CD8+ ratio as a prognostic factor in patients with metastatic melanoma receiving chemoimmunotherapy. J Clin Oncol 14: 1690–1696. 8622089 10.1200/JCO.1996.14.5.1690

[16] Zerbini A, Pilli M, Penna A, Pelosi G, Schianchi C, et al (2006) Radiofrequency thermal ablation of hepatocellular carcinoma liver nodules can activate and enhance tumor-specific T-cell responses. Cancer Res 66: 1139–1146. 16424051 10.1158/0008-5472.CAN-05-2244

[17] Li M, Zhang S, Zhou Y, Guo Y, Jiang X, et al (2010) Argon-helium cryosurgery for treatment of C6 gliomas in rats and its effect on cellular immunity. Technol Cancer Res Treat 9: 87–94. 20082534 10.1177/153303461000900110

[18] Wu F, Wang ZB, Lu P, Xu ZL, Chen WZ, et al (2004) Activated anti-tumor immunity in cancer patients after high intensity focused ultrasound ablation. Ultrasound Med Biol 30: 1217–1222. 15550325 10.1016/j.ultrasmedbio.2004.08.003

[19] Naumnik W, Chyczewska E, Kovalchuk O, Talalaj J, Izycki T, et al (2004) Serum levels of interleukin-18 (IL-18) and soluble interleukin-2 receptor (sIL-2R) in lung cancer. Rocz Akad Med Bialymst 49: 246–251. 15631351

[20] Brivio F, Lissoni P, Mancini D, Tisi E, Tancini G, et al (1991) Effect of antitumor surgery on soluble interleukin-2 receptor serum levels. Am J Surg 161: 466–469. 1827961 10.1016/0002-9610(91)91113-w

[21] Murakami S, Sakata H, Tsuji Y, Okubo K, Hamada S, et al.. (2002) Serum soluble interleukin-2 receptor as a predictor of lymph node metastasis in early gastric cancer. Dig Surg 19: 9–13; discussion 14. 10.1159/000051999 11961349

[22] Sakata H, Murakami S, Hirayama R (2002) Serum soluble interleukin-2 receptor (IL-2R) and immunohistochemical staining of IL-2R/Tac antigen in colorectal cancer. Int J Clin Oncol 7: 312–317. 12402066 10.1007/s101470200046

[23] Howell WM, Rose-Zerilli MJ (2006) Interleukin-10 polymorphisms, cancer susceptibility and prognosis. Fam Cancer 5: 143–149. 16736283 10.1007/s10689-005-0072-3

[24] Langsenlehner U, Krippl P, Renner W, Yazdani-Biuki B, Eder T, et al (2005) Interleukin-10 promoter polymorphism is associated with decreased breast cancer risk. Breast Cancer Res Treat 90: 113–115. 15803357 10.1007/s10549-004-3607-7

[25] Fiorentino DF, Zlotnik A, Vieira P, Mosmann TR, Howard M, et al (1991) IL-10 acts on the antigen-presenting cell to inhibit cytokine production by Th1 cells. J Immunol 146: 3444–3451. 1827484

[26] Carter NA, Vasconcellos R, Rosser EC, Tulone C, Munoz-Suano A, et al (2011) Mice lacking endogenous IL-10-producing regulatory B cells develop exacerbated disease and present with an increased frequency of Th1/Th17 but a decrease in regulatory T cells. J Immunol 186: 5569–5579. 21464089 10.4049/jimmunol.1100284

[27] Huss DJ, Winger RC, Peng H, Yang Y, Racke MK, et al (2010) TGF-beta enhances effector Th1 cell activation but promotes self-regulation via IL-10. J Immunol 184: 5628–5636. 20393141 10.4049/jimmunol.1000288 PMC3804066

[28] Yao Z (2004) Adaptive immune response cells: T lymphocyte. In: CHEN WF, Jin BQ, editors. Immunology. 100–109.

[29] Sanchez-Ortiz RF, Tannir N, Ahrar K, Wood CG (2003) Spontaneous regression of pulmonary metastases from renal cell carcinoma after radio frequency ablation of primary tumor: an in situ tumor vaccine? J Urol 170: 178–179. 12796677 10.1097/01.ju.0000070823.38336.7b

[30] Gravante G, Sconocchia G, Ong SL, Dennison AR, Lloyd DM (2009) Immunoregulatory effects of liver ablation therapies for the treatment of primary and metastatic liver malignancies. Liver Int 29: 18–24. 19018971 10.1111/j.1478-3231.2008.01915.x

[31] Wu F, Wang ZB, Cao YD, Zhou Q, Zhang Y, et al (2007) Expression of tumor antigens and heat-shock protein 70 in breast cancer cells after high-intensity focused ultrasound ablation. Ann Surg Oncol 14: 1237–1242. 17187168 10.1245/s10434-006-9275-6

[32] Dromi SA, Walsh MP, Herby S, Traughber B, Xie J, et al (2009) Radiofrequency ablation induces antigen-presenting cell infiltration and amplification of weak tumor-induced immunity. Radiology 251: 58–66. 19251937 10.1148/radiol.2511072175 PMC2663580

[33] Nijkamp MW, Borren A, Govaert KM, Hoogwater FJ, Molenaar IQ, et al (2010) Radiofrequency ablation of colorectal liver metastases induces an inflammatory response in distant hepatic metastases but not in local accelerated outgrowth. J Surg Oncol 101: 551–556. 20461760 10.1002/jso.21570

[34] Wissniowski TT, Hansler J, Neureiter D, Frieser M, Schaber S, et al (2003) Activation of tumor-specific T lymphocytes by radio-frequency ablation of the VX2 hepatoma in rabbits. Cancer Res 63: 6496–6500. 14559842

[35] Widenmeyer M, Shebzukhov Y, Haen SP, Schmidt D, Clasen S, et al (2011) Analysis of tumor antigen-specific T cells and antibodies in cancer patients treated with radiofrequency ablation. Int J Cancer 128: 2653–2662. 20715115 10.1002/ijc.25601

[36] Liu JG, Chen FL, Ge CL, Gong MY, Zuo HB, et al (2011) Cryosurgery for treatment of subcutaneously xenotransplanted tumors in rats and its effect on cellular immunity. Technol Cancer Res Treat 10: 339–346. 21728391 10.7785/tcrt.2012.500211

[37] Li M, Liu J, Zhang SZ, Zhou Y, Guo YW, et al (2011) Cellular immunologic response to primary cryoablation of C6 gliomas in rats. Technol Cancer Res Treat 10: 95–100. 21214292 10.7785/tcrt.2012.500183

[38] Fietta AM, Morosini M, Passadore I, Cascina A, Draghi P, et al (2009) Systemic inflammatory response and downmodulation of peripheral CD25+Foxp3+ T-regulatory cells in patients undergoing radiofrequency thermal ablation for lung cancer. Hum Immunol 70: 477–486. 19332094 10.1016/j.humimm.2009.03.012

[39] Zhou L, Fu JL, Lu YY, Fu BY, Wang CP, et al (2010) Regulatory T cells are associated with post-cryoablation prognosis in patients with hepatitis B virus-related hepatocellular carcinoma. J Gastroenterol 45: 968–978. 20411280 10.1007/s00535-010-0243-3

[40] Haen SP, Pereira PL, Salih HR, Rammensee HG, Gouttefangeas C (2011) More than just tumor destruction: immunomodulation by thermal ablation of cancer. Clin Dev Immunol 2011: 160250. 22242035 10.1155/2011/160250 PMC3254009

[41] Machlenkin A, Goldberger O, Tirosh B, Paz A, Volovitz I, et al (2005) Combined dendritic cell cryotherapy of tumor induces systemic antimetastatic immunity. Clin Cancer Res 11: 4955–4961. 16000595 10.1158/1078-0432.CCR-04-2422

[42] Clasen S, Krober SM, Kosan B, Aebert H, Fend F, et al (2008) Pathomorphologic evaluation of pulmonary radiofrequency ablation: proof of cell death is characterized by DNA fragmentation and apoptotic bodies. Cancer 113: 3121–3129. 18973180 10.1002/cncr.23882

[43] Hoffmann NE, Bischof JC (2002) The cryobiology of cryosurgical injury. Urology 60: 40–49. 12206847 10.1016/s0090-4295(02)01683-7

[44] Lee EW, Wong D, Prikhodko SV, Perez A, Tran C, et al (2012) Electron microscopic demonstration and evaluation of irreversible electroporation-induced nanopores on hepatocyte membranes. J Vasc Interv Radiol 23: 107–113. 22137466 10.1016/j.jvir.2011.09.020

[45] Lee EW, Chen C, Prieto VE, Dry SM, Loh CT, et al (2010) Advanced hepatic ablation technique for creating complete cell death: irreversible electroporation. Radiology 255: 426–433. 20413755 10.1148/radiol.10090337

[46] Miller L, Leor J, Rubinsky B (2005) Cancer cells ablation with irreversible electroporation. Technol Cancer Res Treat 4: 699–705. 16292891 10.1177/153303460500400615

[47] Maor E, Ivorra A, Mitchell JJ, Rubinsky B (2010) Vascular smooth muscle cells ablation with endovascular nonthermal irreversible electroporation. J Vasc Interv Radiol 21: 1708–1715. 20933436 10.1016/j.jvir.2010.06.024 PMC2966522

[48] Neal RE, 2nd, Singh R, Hatcher HC, Kock ND, Torti SV, et al (2010) Treatment of breast cancer through the application of irreversible electroporation using a novel minimally invasive single needle electrode. Breast Cancer Res Treat 123: 295–301. 20191380 10.1007/s10549-010-0803-5 PMC3021965

[49] Schoellnast H, Monette S, Ezell PC, Deodhar A, Maybody M, et al (2011) Acute and subacute effects of irreversible electroporation on nerves: experimental study in a pig model. Radiology 260: 421–427. 21642418 10.1148/radiol.11103505 PMC6939978

[50] Dupuy DE, Aswad B, Ng T (2011) Irreversible electroporation in a Swine lung model. Cardiovasc Intervent Radiol 34: 391–395. 21191587 10.1007/s00270-010-0091-9

[51] Gazzaniga S, Bravo A, Goldszmid SR, Maschi F, Martinelli J, et al (2001) Inflammatory changes after cryosurgery-induced necrosis in human melanoma xenografted in nude mice. J Invest Dermatol 116: 664–671. 11348453 10.1046/j.0022-202x.2001.01313.x

[52] Mirabello L, Troisi RJ, Savage SA (2009) Osteosarcoma incidence and survival rates from 1973 to 2004: data from the Surveillance, Epidemiology, and End Results Program. Cancer 115: 1531–1543. 19197972 10.1002/cncr.24121 PMC2813207

[53] Link MP, Goorin AM, Miser AW, Green AA, Pratt CB, et al (1986) The effect of adjuvant chemotherapy on relapse-free survival in patients with osteosarcoma of the extremity. N Engl J Med 314: 1600–1606. 3520317 10.1056/NEJM198606193142502

